# Evidence for broad cross-reactivity of the SARS-CoV-2 NSP12-directed CD4^+^ T-cell response with pre-primed responses directed against common cold coronaviruses

**DOI:** 10.3389/fimmu.2023.1182504

**Published:** 2023-05-05

**Authors:** Tim Westphal, Maria Mader, Hendrik Karsten, Leon Cords, Maximilian Knapp, Sophia Schulte, Lennart Hermanussen, Sven Peine, Vanessa Ditt, Alba Grifoni, Marylyn Martina Addo, Samuel Huber, Alessandro Sette, Marc Lütgehetmann, Sven Pischke, William W. Kwok, John Sidney, Julian Schulze zur Wiesch

**Affiliations:** ^1^ Infectious Diseases Unit I, Department of Medicine, University Medical Center Hamburg-Eppendorf, Hamburg, Germany; ^2^ German Center for Infection Research Deutsches Zentrum für Infektionsforschung (DZIF), Partner Site Hamburg-Lübeck-Borstel-Riems, Hamburg, Germany; ^3^ Institute of Transfusion Medicine, University Medical Center Hamburg-Eppendorf, Hamburg, Germany; ^4^ Center for Infectious Disease and Vaccine Research, La Jolla Institute for Immunology (LJI), La Jolla, CA, United States; ^5^ Department for Clinical Immunology of Infectious Diseases, Bernhard Nocht Institute for Tropical Medicine, Hamburg, Germany; ^6^ Institute of Infection Research and Vaccine Development, University Medical Center Hamburg-Eppendorf, Hamburg, Germany; ^7^ Institute of Medical Microbiology, Virology and Hygiene, University Medical Center Hamburg-Eppendorf, Hamburg, Germany; ^8^ Benaroya Research Institute at Virginia Mason, Seattle, WA, United States

**Keywords:** SARS-CoV-2, NSP12, CD4+ T-cells, RNA-dependant RNA polymerase, Cross-reactivities, epitope analysis, sequence identities, common cold coronaviruses

## Abstract

**Introduction:**

The nonstructural protein 12 (NSP12) of the severe acute respiratory syndrome coronavirus type 2 (SARS-CoV-2) has a high sequence identity with common cold coronaviruses (CCC).

**Methods:**

Here, we comprehensively assessed the breadth and specificity of the NSP12-specific T-cell response after *in vitro* T-cell expansion with 185 overlapping 15-mer peptides covering the entire SARS-CoV-2 NSP12 at single-peptide resolution in a cohort of 27 coronavirus disease 2019 (COVID-19) patients. Samples of nine uninfected seronegative individuals, as well as five pre-pandemic controls, were also examined to assess potential cross-reactivity with CCCs.

**Results:**

Surprisingly, there was a comparable breadth of individual NSP12 peptide-specific CD4^+^ T-cell responses between COVID-19 patients (mean: 12.82 responses; range: 0–25) and seronegative controls including pre-pandemic samples (mean: 12.71 responses; range: 0–21). However, the NSP12-specific T-cell responses detected in acute COVID-19 patients were on average of a higher magnitude. The most frequently detected CD4^+^ T-cell peptide specificities in COVID-19 patients were aa236–250 (37%) and aa246–260 (44%), whereas the peptide specificities aa686–700 (50%) and aa741–755 (36%), were the most frequently detected in seronegative controls. In CCC-specific peptide-expanded T-cell cultures of seronegative individuals, the corresponding SARS-CoV-2 NSP12 peptide specificities also elicited responses *in vitro*. However, the NSP12 peptide-specific CD4^+^ T-cell response repertoire only partially overlapped in patients analyzed longitudinally before and after a SARS-CoV-2 infection.

**Discussion:**

The results of the current study indicate the presence of pre-primed, cross-reactive CCC-specific T-cell responses targeting conserved regions of SARS-CoV-2, but they also underline the complexity of the analysis and the limited understanding of the role of the SARS-CoV-2 specific T-cell response and cross-reactivity with the CCCs.

## 1 Introduction

The severe acute respiratory syndrome coronavirus type 2 (SARS-CoV-2) is the virus responsible for the ongoing pandemic with extensive global implications, and infection leads to the coronavirus disease 2019 (COVID-19), which presents as a flu-like illness and is divided into different severity levels by the WHO, ranging from asymptomatic through clinical progression levels up to death due to the disease (1).

SARS-CoV-2 is a large single-strand positive RNA virus that encodes four structural proteins (spike glycoprotein, envelope, mebrane, and nucleoprotein), nine accessory proteins, and 16 nonstructural proteins (NSPs), resulting in a total number of at least 29 proteins. It has been shown that exposure to structural proteins can elicit a virus-specific CD4^+^ T-cell response that varies in magnitude and breadth (2, 3).

The RNA-dependent RNA polymerase (RdRp), which is encoded by the *NSP12* gene, consists of 932 amino acids and is crucial for the replication of the virus. This genomic region is highly conserved, as evidenced by the sequence similarity with other Coronaviridae (4, 5). The NSP12 of SARS-CoV-2 has a higher sequence identity with common cold coronaviruses (CCCs) than, for example, the spike glycoprotein (6, 7). After the assembly with the co-factors NSP7 and NSP8, the functional polymerase fulfills its task of replicating the SARS-CoV-2 genome (8). The NSP12 "without has protein" has an essential role in the life cycle of the virus, and NSP12 is the target of the antiviral nucleoside analog inhibitor remdesivir (9).

Recently, it has been shown that there might be complex immunological interactions between CCCs (HKU1, NL63, 229E, OC43) and the SARS-CoV-2-specific immune response, potentially altering the clinical course of COVID-19 (10–16).

We and others have previously mapped the breadth and specificity of the spike-specific CD4^+^ T-cell response (3, 17), as well as N-, E-, and M-specific CD4^+^ T-cell responses (2, 18). In the current study, we characterized the NSP12-specific T-cell response in COVID-19 patients with an overlapping 15-mer peptide set. Furthermore, we examined the potential SARS-CoV-2 NSP12-specific cross-reactivity with other corresponding CCC proteins with pronounced sequence identities to gain further insight into SARS-CoV-2-specific T-cell responses.

## 2 Materials and methods

### 2.1 Ethics statement

All study participants gave written informed consent. The study was conducted in accordance with the Declaration of Helsinki and approved by the local ethics board of the Ärztekammer Hamburg (PV4780, PV7298).

### 2.2 Patient cohort

Study participants were recruited at the University Medical Center Hamburg-Eppendorf between May and December 2021. The “acutely infected” group comprised patients hospitalized with COVID-19 infection who were admitted to the infectious diseases ward. An infection with SARS-CoV-2 was confirmed by polymerase chain reaction (PCR) from oropharyngeal and/or nasopharyngeal swabs, as previously described (19). “Acute COVID-19 patients” were defined as being hospitalized due to a SARS-CoV-2 infection with a maximum of 2 months between the date of diagnosis and blood sampling. HH-N12-8, who was acutely ill with COVID-19 and had blood drawn after 2 months, was still viremic at the time of collection.

The “resolved COVID-19 patients” group, defined as patients who tested positive for COVID-19 but have since recovered and were moved out of isolation, included medical and nonmedical staff of the University Medical Center Hamburg-Eppendorf and associated institutions. A resolved SARS-CoV-2 infection was confirmed by a previous positive PCR result and/or positive SARS-CoV-2 nucleocapsid (NP) antibodies and a history of acute flu-like illness. The time since infection in this group ranged between 11 and 448 days (average: 102 days).

### 2.3 Seronegative and pre-pandemic controls

Seronegative controls were individuals who were recruited when fewer than 5% of the general population had been infected with COVID-19 and were defined as (A) NP seronegative, (B) neither believably having a history of flu-like symptoms since the beginning of the pandemic, nor (C) having ever been tested SARS-CoV-2 PCR positive. Pre-pandemic samples were defined as peripheral blood mononuclear cells (PBMCs) that were frozen and stored before 01 January 2020.

### 2.4 Nonstructural protein 12 peptides

In total, 15-mer peptides overlapping by 10 amino acids and corresponding to the complete NSP12 amino acid sequence were synthesized (peptides and elephants, Hennigsdorf, Germany). The complete amino acid sequence is depicted in **Supplementary Table S1**. All peptides were divided into four pools of either 46 or 47 peptides. For *in vitro* culture, peptide pools were used at a concentration of 10 μg/ml per single peptide. For the enzyme-linked immunospot assay (ELISpot), the final concentration of every single peptide was 10 μg/ml.

### 2.5 CCC peptides

A total of 18 15-mer peptides corresponding to the CCC (HKU1, NL63, 229E) NSP12 amino acid sequences were synthesized (peptides and elephants, Hennigsdorf, Germany). Because none of the seronegative controls included in the CCC cross-reactivity experiment tested positive for OC43, we did not order 15-mer peptides corresponding to the OC43 sequence. Canonical protein amino acid sequences of CCCs were extracted from the reviewed UniProtKB database (20). Six different peptide specificities were produced, and each specificity was generated in variants matching the amino acids specific to each of the three CCCs (**Supplementary Table S1**).

### 2.6 Sample processing and T-cell expansion

Venous whole blood samples from the study participants were collected in a BD Vacutainer^®^ CPT™ (Becton Dickinson GmbH, Heidelberg, Germany). PBMCs were isolated by centrifugation and used fresh. Frozen PBMCs of pre-pandemic samples, acquired before 01 January 2020, were thawed. In 24-well culture plates, 30–50 × 10^6^ PBMCs were cultured per patient in Roswell Park Memorial Institute medium (RPMI) supplemented with 10% fetal calf serum, penicillin, and streptomycin (R10).

The T-cell expansion was induced in duplicates by stimulation with one of the four peptide pools consisting of overlapping 15-mer peptides covering the whole SARS-CoV-2 NSP12 at 10 µg/ml, anti-CD28/anti-CD49d co-stimulation, and 50 U/ml recombinant interleukin 2 (rIL-2) at 37°C and 5% CO_2_. When necessary, 50 U/ml rIL-2 and R10 were used for exchanges of medium. After 11–13 days, the cells were harvested and used for the T-cell assays described below.

### 2.7 IFN-γ EliSpot assay

IFN-γ-ELISpot assays were performed as described before (2). In short, approximately 100,000 pre-cultured cells were distributed into each well of 96-well plates pre-coated with IFN-γ antibodies (clone 1-D1K, Mabtech AB, Nacka Strand, Sweden). The cells were then separately stimulated with each of the 46 or 47 peptides from the corresponding peptide pool at a concentration of 10 µg/ml for 18–20 h at 37°C and 5% CO_2_. Anti-CD3-stimulated cells served as a positive control, and unstimulated cells in R10 medium served as a negative control.

After a washing step, IFN-γ was detected with a biotinylated anti-IFN-γ antibody (clone 7-B6-1; Mabtech AB, Nacka Strand, Sweden), alkaline phosphatase-conjugated streptavidin, and a 5-bromo-4-chloro-3-indolyl phosphate (BCIP)/nitroblue tetrazolium (NBT) substrate solution. Results were considered positive if a single peptide well showed at least three times the number of IFN-γ spots compared to the corresponding control well.

### 2.8 Intracellular cytokine staining

Positive results in the ELISpot assay were validated by intracellular cytokine staining (ICS) for IFN-γ, as described previously (2). The pre-cultured cells were re-stimulated with the peptides showing a positive result at a concentration of 10 µg/ml for 16 h at 37°C and 5% CO_2_. One negative control per pool consisting of cells and R10 medium only and a positive control per patient stimulated with phorbol-12-myristate-13-acetate and ionomycin (10 µg/ml) were also set up. After 1 h, 5 µl/ml of Brefeldin A (Sigma-Aldrich, St. Louis, MO, USA) solution was added to inhibit cytokine secretion.

The cells were stained with Zombie NIR fixable viability dye (BioLegend, San Diego, CA, USA) per the manufacturer’s instructions and the following fluorochrome-conjugated monoclonal antibodies on the cell surface: anti-CD3 (clone UCHT1, AlexaFluor700), anti-CD4 (clone SK3, BV510), anti-CD8 (clone RPA-T8, PerCP-Cy5.5), anti-CD14 (clone 63D3, APC-Cy7), and anti-CD19 (clone HIB19, APC-Cy7). After fixation and permeabilization of the cells using the FoxP3 transcription factor staining buffer set (eBioscience, Thermo Fisher Scientific), the cells were stained for intracellular IFN-γ using a monoclonal anti-IFN-γ antibody (clone 4S.B3, PE-Dazzle594). All monoclonal antibodies were purchased from BioLegend.

We defined a T-cell response as positive under three conditions: a percentage of CD4^+^ or CD8^+^ T cells positive for the secretion of IFN-γ three times higher than the negative control, at least 0.02% IFN-γ-positive cells, and if the population could be visibly separated from the negative control. The cells were acquired on an LSRFortessa II cytometer (BD Biosciences) using FACSDiva version 8 for Windows (BD Biosciences). The gating strategy is shown in **Supplementary Figure 1**.

### 2.9 HLA typing

High-definition molecular HLA class I and II typing from whole blood samples was performed for 17 individuals at the Institute of Transfusion Medicine at the University Medical Center Hamburg-Eppendorf by PCR sequence-specific oligonucleotide (PCR-SSO) technique using the commercial kit SSO LabType (One Lambda, Canoga Park, CA, USA), as previously described (21). The gating strategy is shown in **Supplementary Figure 1**.

### 2.10 SARS-CoV-2 and CCC serologies

Antibody levels were determined by the Roche Elecsys SARS-CoV-2 S assay in arbitrary units (AU) per milliliter as described previously (22) with a linear range from 0.4 to 25,000 AU/ml. A negative test result was defined as a result <0.8 AU/ml, a low positive response between 0.8 and 103 AU/ml, and a positive response >103 AU/ml. SARS-CoV-2 nucleocapsid antibodies were assessed by the Elecsys anti-NC-SARS-CoV-2 Ig assay (Roche, Mannheim, Germany; cutoff: ≥ 1 COI/ml). Serologies for the CCCs HKU1, NL63, 229E, and OC43 were available for 14 individuals by a line blot assay using the recomLine SARS-CoV-2 IgG kit (MIKROGEN GmbH, Neuried, Bavaria, Germany) as previously described (23).

### 2.11 *Ex vivo* ICS

*Ex vivo* ICS was performed as previously described (3). In short, cryopreserved PBMCs from COVID-19 patients were stimulated with an NSP12 Best-Of peptide pool consisting of 11 peptides that elicited responses in most of the previously studied patients. The cells were then washed and stained with Zombie NIR fixable viability dye (BioLegend) and fluorochrome-labeled monoclonal antibodies targeting CD3 (clone UCHT1, AlexaFluor700), CD4 (clone RPA-T4, BV785), CD8 (clone RPA-T8, BV650), CD14 (clone 63D3, APC-Cy7), and CD19 (clone HIB19, APC-Cy7).

After fixation and permeabilization of the cells using the FoxP3 transcription factor staining buffer set (eBioscience, Thermo Fischer Scientific), the cells were stained for intracellular IFN-γ using a monoclonal anti-IFN-γ antibody (clone 4S.B3, PE-Dazzle594).

### 2.12 HLA (MHC class II) binding capacity

*In vitro* HLA binding assays with 14 peptides that frequently elicit NSP12-specific CD4^+^ T-cell responses were performed using purified HLA-class II molecules, as previously described (24). Worldwide population coverage at the DRB1 locus afforded by each epitope was predicted using the population coverage tool hosted by the IEDB (25, 26). These data are based on allele frequency data provided by The Allele Frequency Net Database (27). Coverage of an allele was considered based on a corresponding binding affinity of 1,000 nM or lower, a binding threshold associated with >80% of known CD4^+^ epitopes for their reported HLA-restricting molecule (28). For this purpose, coverage estimates imply, but cannot confirm T-cell recognition, and thus may be overestimated. Conversely, because coverage estimates only consider alleles for which binding has been examined experimentally, they may also underestimate coverage.

### 2.13 Data analysis and statistics

The analysis of all flow cytometric data was performed in FlowJo version 10 for Windows (Treestar, Ashland, OR, USA). All graphs and statistical analyses were conducted in GraphPad Prism version 7.0 for Windows (GraphPad Software Inc., San Diego, CA, USA). Data are visualized as the mean with a standard deviation. The following tests for statistical significance were used: the Mann–Whitney *U* test (for testing of two groups) and Kruskal–Wallis and ANOVA with Dunn’s correction for multiple analyses (for testing of three or more groups). For all tests, two-tailed *p*-values were generated. Results with a *p*-value less or equal to 0.05 were considered statistically significant. (levels of significance: ^*^ *p* < 0.05; ^**^ *p* < 0.01; ^***^ *p* < 0.001; and ^****^ *p* < 0.0001).

## 3 Results

### 3.1 Clinical features of the study cohort

The demographic and clinical characteristics of the patients are outlined in **Table 1**. The study cohort consisted of 27 patients with a SARS-CoV-2 infection, of whom we were able to collect (A) PBMCs early during SARS-CoV-2 infection (*n* = 15) or patients or (B) at the stage of resolved infection in 12 individuals (1–15 months after the infection). The study included 19 male and eight female patients, with a mean age of 49.2 years (range: 19–95).

**Table 1:** Study cohort characteristics including demographical and clinical data.

	Acute COVID-19 infection	Resolved COVID-19 infection	Seronegative controls	Pre-pandemic controls
	*n* = 15	*n* = 12	*n* = 9	*n* = 5
**Age in years (range)**	61 (19–95)	37.4 (21–63)	25.3 (21–34)	–
Sex at birth				
Female (%)	4 (26.7%)	4 (33.3%)	3 (33.3%)	–
Male (%)	11 (73.3%)	8 (66.7%)	6 (66.7%)	–
Unknown (%)	–	–	–	5 (100%)
Disease severity				
Uninfected—WHO 0 (%)	–	–	9 (100%)	5 (100%)
Ambulatory mild disease—WHO 1–3 (%)	3 (20%)	8 (66.7%)	–	–
Hospitalized: moderate disease—WHO 4–5 (%)	5 (33.3%)	1 (8.3%)	–	–
Hospitalized: severe disease—WHO 6–9 (%)	7 (46.7%)	2 (16.7%)	–	–
Unknown	–	1 (8.3%)	–	–

According to the WHO severity classification, 11 (41%) of the patients had ambulatory mild disease. Six (22%) patients were hospitalized with a moderate disease and nine (33%) patients with a severe disease. The detailed clinical characteristics can be found in **Supplementary Table S2**.

Furthermore, PBMC samples from 14 individuals without a history of SARS-CoV-2 infection were included. They were substratified into seronegatives (*n* = 9) (without a history of COVID-19) and pre-pandemic controls (*n* = 5). There were no characteristics available on the pre-pandemic controls because they were anonymous buffy coats from healthy blood donors.

### 3.2 Similar breadth of the NSP12-specific CD4^+^ T-cell response regardless of the infection status, but a higher magnitude of the T-cell response in acute COVID-19 patients

In the current study, we assessed the breadth of the virus-specific T-cell response and its specificities within the SARS-CoV-2 NSP12 on a single-peptide level in patients with acute and resolved COVID-19. As described earlier, *ex vivo* ELISpot assays after stimulation with single peptides and NSP12 peptide pools showed a low overall IFN-γ response with a magnitude barely above the limit of detection of this assay (data not shown).

**Figure 1:**
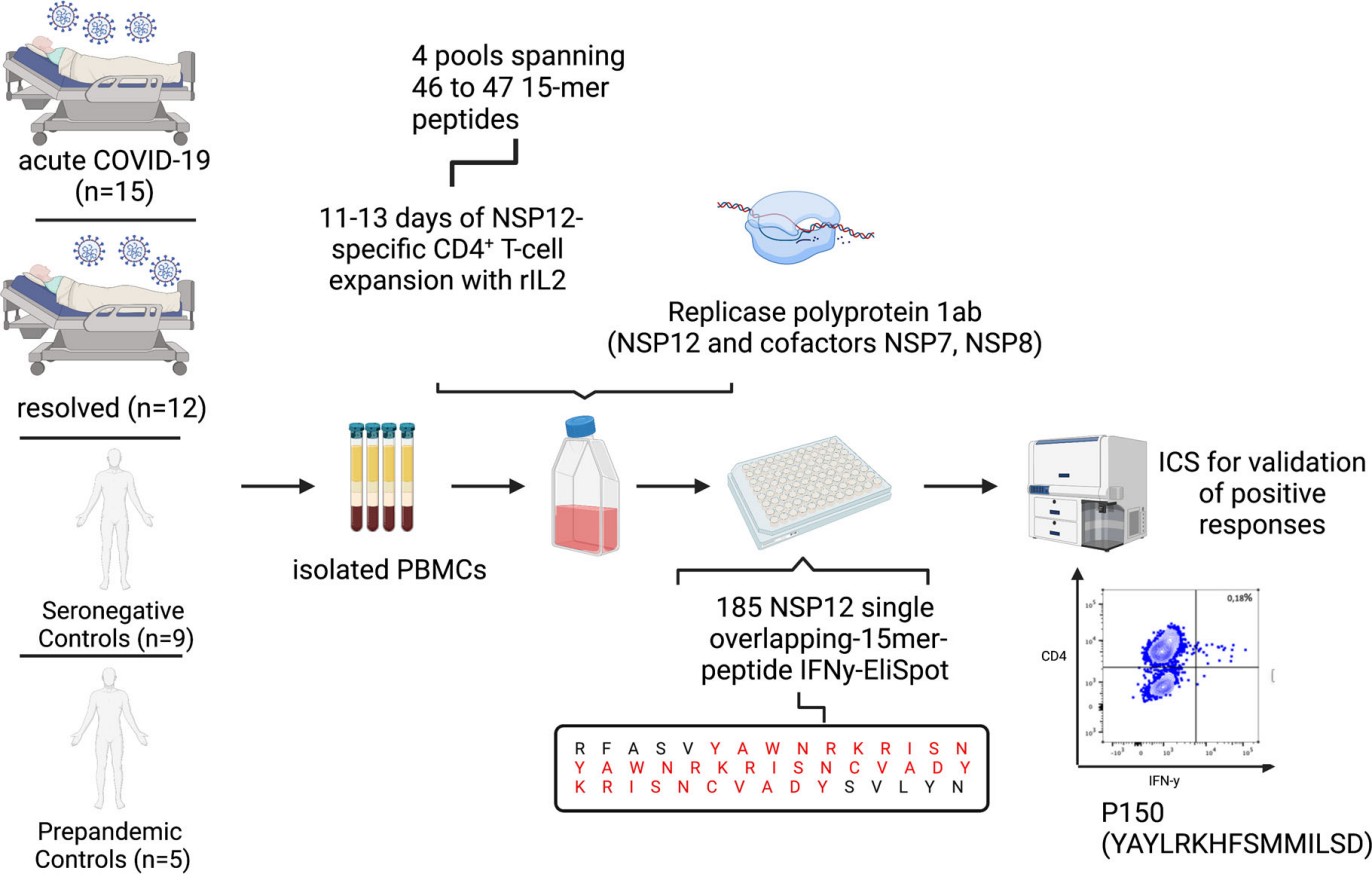
Experimental setup of the 15-mer single-peptide IFN-γ-ELISpot after 11–13 days of *in vitro* peptide-specific culture with different peptide pools, each spanning 46 to 47 peptides, and ICS after single-peptide re-stimulation for validation of positive peptide-specific T-cell responses.

**Figure 2:**
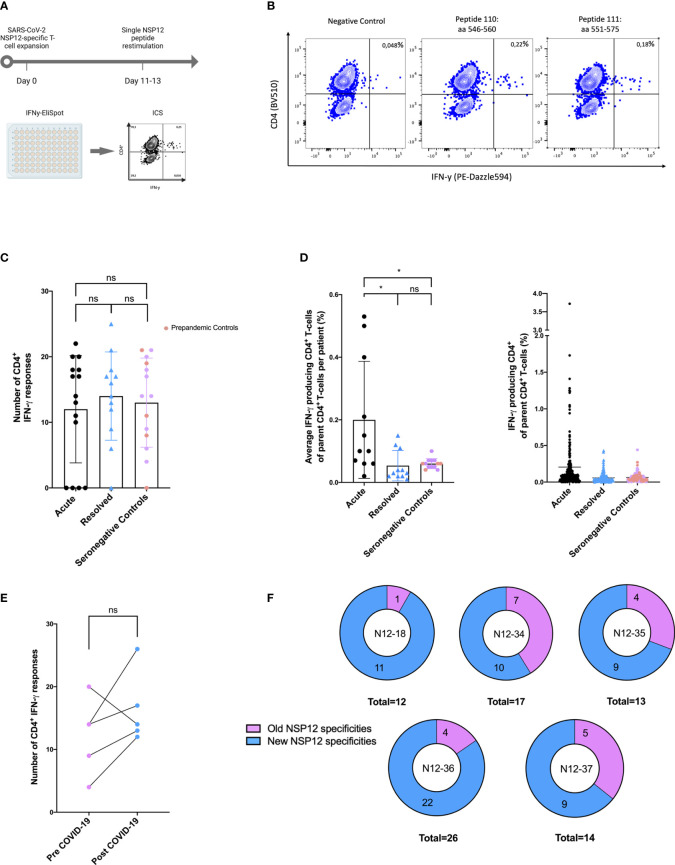
**(A–F)** SARS-CoV-2 seronegative individuals demonstrated highly cross-reactive NSP12-specific CD4^+^ T-cell responses with low IFN-γ magnitude comparable to post-acute SARS-CoV-2 infection. **(A)** NSP12-specific T-cells were expanded *in vitro* with pools of overlapping NSP12 peptides in the presence of anti-CD28/anti-CD49d antibodies and IL-2. After 11–13 days, the cells were analyzed for IFN-γ production by ELISpot and validated by intracellular cytokine staining after restimulation with single peptides. **(B)** CD4^+^ T-cells predominated NSP12-specific IFN-γ responses in all groups. Representative flow cytometry plots of HH-N12-7 with acute SARS-CoV-2 infection. **(C)** Comparable numbers of recognized NSP12 peptides between individuals with acute or resolved SARS-CoV-2 infection and seronegative controls indicated relevant cross-reactivity of previously primed CD4^+^ T-cells. **(D)** The magnitude of IFN-γ production of NSP12-specific CD4^+^ T-cells in individuals with resolved SARS-CoV-2 infection and seronegative individuals compared to individuals with acute infection. **(E)** The breadth of cross-reactive NSP12-specific CD4^+^ T-cell responses in seronegative individuals is boosted by SARS-CoV-2 infection. **(F)** Increased breadth derives mostly from newly recognized specificities. Data are expressed as mean with a standard deviation. Mann–Whitney *U* test was performed to assess statistical significance. ns < 0.05; ^*^ *p* < 0.05.

Next, we investigated the T-cell responses, after *in vitro* NSP12 peptide-specific cell culture using four pools of 46 to 47 peptides and re-stimulating with single 15-mer peptides of the SARS-CoV-2 NSP12 using IFN-g ELISpot after 11–13 days (**Figure 1**). Each positive ELISpot response was confirmed and classified as a CD4^+^ or CD8^+^ T-cell response by intracellular cytokine staining (ICS) for IFN-γ after re-stimulation with the respective single peptide (**Figure 2**). Representative flow-cytometric plots for NSP12-specific CD4^+^ T-cell responses are shown in **Figure 2**, and representative plots for CD8^+^ T-cell responses are shown in **Supplementary Figure S2**.

The majority of the elicited IFN-γ responses proved to be CD4^+^ T-cell responses in the flow-cytometric analysis. Of the COVID-19 patients, 81% (22 out of 27) and 92% (13 out of 14) of the seronegative controls showed peptide-specific CD4^+^ T-cell responses to at least one NSP12 peptide specificity. Five COVID-19 patients, four of them acutely ill and one recovered after a SARS-CoV-2 infection, did not show any responses, three of whom were receiving immunosuppressive medication at the time of blood sampling.

Altogether, there were 348 CD4^+^ T-cell responses detected in 27 COVID-19 patients and 178 responses in 14 seronegative controls. The detailed response pattern can be found in **Supplementary Table S3**. Somewhat unexpectedly, the number of NSP12-specific CD4^+^ T-cell responses directed against individual peptides in an individual did not significantly differ between COVID-19 patients (mean: 12.82 responses; range: 0–25; *p* > 0.05) and seronegative or pre-pandemic controls (mean: 12.71 responses; range: 0–21) (**Figure 2**).

However, the average magnitude of the individual peptide-specific responses was significantly higher in acute COVID-19 patients (mean: 0.2%; range: 0.02–0.53) compared to individuals after a recovered SARS-CoV-2 infection (mean: 0.05%; range: 0.02–0.15) or individuals without previous exposure to SARS-CoV-2 (mean: 0.06%; range: 0.04–0.1; ^*^ *p* < 0.05). We found no statistically significant difference in the number of detected peptides between patients with a resolved COVID-19 infection and seronegative controls (**Figure 2**).

In either disease status, 160 out of 185 (86%) 15-mer overlapping peptides elicited a CD4^+^ T-cell response in at least one individual.

The peptide specificities NSP12_48 (aa236–250) and NSP12_50 (aa246–260) were each recognized by more than 35% of patients with an ongoing or a resolved SARS-CoV-2 infection (**Table 2**). Furthermore, we were able to define nine peptides that each elicited a CD4^+^ T-cell response in more than 25% of individuals (**Table 2**) in our SARS-CoV-2 seronegative cohort. Of interest, the peptide specificity aa686–700 was recognized by 50% of the seronegative controls and by 22% of the COVID-19 patients. It showed sequence identity with the corresponding sequences of the CCCs of up to 90% (**Table 3**).

**Table 2:** Most frequently detected peptides in COVID-19 patients and seronegative individuals.

Peptide	aa position	Sequence															RF
COVID-19 patients: most frequently detected peptides of the NSP12																	
NSP12_48	236–250	S	Y	Y	S	L	L	M	P	I	L	T	L	T	R	A	37%
NSP12_49	241–255	L	M	P	I	L	T	L	T	R	A	L	T	A	E	S	30%
NSP12_50	246–260	T	L	T	R	A	L	T	A	E	S	H	V	D	T	D	44%
NSP12_106	526–540	A	L	F	A	Y	T	K	R	N	V	I	P	T	I	T	22%
NSP12_129	641–655	K	H	T	T	C	C	S	L	S	H	R	F	Y	R	L	26%
NSP12_130	646–660	C	S	L	S	H	R	F	Y	R	L	A	N	E	C	A	26%
NSP12_131	651–665	R	F	Y	R	L	A	N	E	C	A	Q	V	L	S	E	26%
NSP12_138	686–700	T	T	A	Y	A	N	S	V	F	N	I	C	Q	A	V	22%
NSP12_139	691–705	N	S	V	F	N	I	C	Q	A	V	T	A	N	V	N	22%
NSP12_149	741–755	F	V	N	E	F	Y	A	Y	L	R	K	H	F	S	M	30%
NSP12_170	846–860	D	I	V	K	T	D	G	T	L	M	I	E	R	F	V	26%
Seronegative controls: most frequently detected peptides of the NSP12																	
NSP12_48	236–250	S	Y	Y	S	L	L	M	P	I	L	T	L	T	R	A	29%
NSP12_125	621–635	K	C	D	R	A	M	P	N	M	L	R	I	M	A	S	29%
NSP12_131	651–665	R	F	Y	R	L	A	N	E	C	A	Q	V	L	S	E	29%
NSP12_135	671–685	G	S	L	Y	V	K	P	G	G	T	S	S	G	D	A	29%
NSP12_138	686–700	T	T	A	Y	A	N	S	V	F	N	I	C	Q	A	V	50%
NSP12_139	691–705	N	S	V	F	N	I	C	Q	A	V	T	A	N	V	N	29%
NSP12_149	741–755	F	V	N	E	F	Y	A	Y	L	R	K	H	F	S	M	36%
NSP12_154	766–780	F	N	S	T	Y	A	S	Q	G	L	V	A	S	I	K	29%
NSP12_170	846–860	D	I	V	K	T	D	G	T	L	M	I	E	R	F	V	36%

**Table 3:** SARS-CoV-2 NSP12 peptide sequence identity with CCCs of the three most frequently detected peptides in seronegative and pre-pandemic controls.

Virus	Sequence														
NSP12_138 (aa686–700)															
SARS-CoV-2	T	T	A	Y	A	N	S	V	F	N	I	C	Q	A	V
229 E	•	•	•	•	•	•	•	•	•	•	•	F	•	•	•
NL63	S	•	•	•	•	•	•	I	•	•	•	F	•	•	•
OC43	•	•	•	F	•	•	•	•	•	•	•	•	•	•	•
HKU1	•	•	•	F	•	•	•	•	•	•	•	•	•	•	•
NSP12_149 (aa741–755)															
SARS-CoV-2	F	V	N	E	F	Y	A	Y	L	R	K	H	F	S	M
229 E	•	•	D	D	•	•	G	•	•	Q	•	•	•	•	•
NL63	•	I	D	D	Y	•	G	•	•	•	•	•	•	•	•
OC43	•	•	T	•	Y	•	E	F	•	•	•	•	•	•	•
HKU1	•	•	•	•	Y	•	E	F	•	C	•	•	•	•	•
NSP12_170 (aa846–860)															
SARS-CoV-2	D	I	V	K	T	D	G	T	L	M	I	E	R	F	V
229 E	•	•	T	•	•	•	A	V	I	L	L	•	•	Y	•
NL63	•	V	•	•	•	•	A	V	V	L	L	•	•	Y	•
OC43	•	L	L	•	•	•	S	V	•	L	•	•	•	•	•
HKU1	•	L	L	•	•	•	S	V	•	L	•	•	•	•	•

Overall, we found a broadly directed, low-level NSP12-specific CD4^+^ T-cell response in COVID-19 patients, with a higher magnitude in the acutely infected patients. Surprisingly, in pre-pandemic and seronegative samples, we detected a similar range of NSP12-specific CD4^+^ T-cell responses.

### 3.3 HLA binding and prediction of HLA restriction

*In vitro* HLA class II binding assays were performed with a subset of frequently detected SARS-CoV-2 NSP12 peptides (**Supplementary Table S1**). These studies indicated that the peptide specificities aa236–250 and aa246–260 that were most frequently recognized in this study (response frequency: 37% and 44%, respectively) could bind seven or more of the 11 DRB1-HLA molecules tested with an affinity of 1,000 nM or better (**Table 4**). This could imply a broad presentation by multiple HLA specificities and might explain the broad recognition in our cohort.

**Table 4:** *In vitro* and *in silico* HLA binding and HLA predictions.

Peptide	aa sequence	aa position		DRB1*01:01	DRB1*03:01	DRB1*04:01	DRB1*04:05	DRB1*07:01	DRB1*08:02	DRB1*09:01	DRB1*11:01	DRB1*12:01	DRB1*13:02	DRB1*15:01	Alleles bound
48	SYYSLLMPILTLTRA	236–250	*In vitro* (IC_50_ nM)	7.7	9,119	224	147	115	498	1,551	73	1,353	–	23	7
			*In silico* (rank)	0.16	38	1.9	5.6	13	33	2.8	4.2	12.65	61	21	
49	LMPILTLTRALTAES	241–255	*In vitro* (IC_50_ nM)	136	12,112	20	367	15	283	123	37	131	409	37	10
			*In silico* (rank)	5.6	13	6.3	9.7	5	0.37	18	2.2	10.2	26	13	
50	TLTRALTAESHVDTD	246–260	*In vitro* (IC_50_ nM)	2,099	1,644	4.1	99	14	501	59	301	4,486	–	1,293	6
			*In silico* (rank)	33	42	23	23	39	18	29	55	51	84	68	
91	SDYDYYRYNLPTMCD	451–465	*In vitro* (IC_50_ nM)	6,666	–	830	3,278	549	3,511	1,431	18,219	29,644	13,645	1,479	2
			*In silico* (rank)	26	48	6.4	9.9	38	47	11	23	68.5	23	30	
92	YRYNLPTMCDIRQLL	456–470	*In vitro* (IC_50_ nM)	12,010	–	5,315	4,645	1,845	–	1,488	8,942	5,124	–	2,915	0
			*In silico* (rank)	46	26	44	23	56	75	49	29	49.5	59	61	
105	YEDQDALFAYTKRNV	521–535	*In vitro* (IC_50_ nM)	15,983	39,384	3,086	–	35	495	8,573	21	–	–	480	4
			*In silico* (rank)	43	67	41	62	55	47	50	19	38.5	53	22	
106	ALFAYTKRNVIPTIT	526–540	*In vitro* (IC_50_ nM)	7,355	709	3,659	12,985	1.6	190	52	6.6	756	32	770	8
			*In silico* (rank)	28	35	33	48	2.7	9.1	31	3.1	38	7.4	20	
129	KHTTCCSLSHRFYRL	641–655	*In vitro* (IC_50_ nM)	279	916	613	644	56	1,009	286	235	59	1,175	447	9
			*In silico* (rank)	37	33	86	86	15	98	45	51	47.5	80	73	
130	CSLSHRFYRLANECA	646–660	*In vitro* (IC_50_ nM)	233	36,524	108	161	792	3,115	345	504	513	–	950	8
			*In silico* (rank)	3.7	63	4.4	9.8	26	47	32	14	25.5	73	23	
131	RFYRLANECAQVLSE	651–665	*In vitro* (IC_50_ nM)	53	8,410	27	270	279	2,347	48	367	6,324	23,820	611	7
			*In silico* (rank)	8	60	1.7	15	45	26	12	16	34.5	23	48	
137	SSGDATTAYANSVFN	681–695	*In vitro* (IC_50_ nM)	1,799	–	5,182	27,195	3,965	–	197	15,925	22,725	–	567	2
			*In silico* (rank)	63	93	84	74	36	93	59	94	81.5	28	59	
148	DVDTDFVNEFYAYLR	736–750	*In vitro* (IC_50_ nM)	856	28,451	16,886	–	–	2,198	6,954	949	–	–	1,181	2
			*In silico* (rank)	37	18	36	30	48	49	50	65	20.5	35	15	
149	FVNEFYAYLRKHFSM	741–755	*In vitro* (IC_50_ nM)	36,126	–	8,366	–	3,835	828	2,515	70	2,537	–	1,252	2
			*In silico* (rank)	12	43	26	19	31	4.8	20	0.11	16.5	64	4.5	
150	YAYLRKHFSMMILSD	746–760	*In vitro* (IC_50_ nM)	495	5,015	102	318	0.73	138	4.8	7.9	96	755	0.23	10
			*In silico* (rank)	11	21	7.6	9.7	0.88	8	4.4	1.8	9.05	3.1	3	

^*^ *In silico* predictions: the MHCII binding predictions were made on 8 August 2022 using the IEDB analysis resource Consensus tool. REFs: (29, 30).

### 3.4 The breadth and specificity of the NSP12-specific CD8^+^ T-cell response in COVID-19 patients and seronegative controls

The *in vitro* culture assay using 15-mer peptides favors the detection of CD4^+^ T-cell responses; however, we did not want to exclude analysis of NSP12-specific CD8^+^ responses from this study *a priori*. The flow-cytometric analyses identified most of the peptide-specific IFN-γ responses as CD4^+^ T-cell responses.

Generally, there was a less broad NSP12-specific CD8^+^ T-cell response with a low magnitude in the majority of individuals (**Supplementary Figure S2**). We detected a median of nine (range: 0–25) NSP12-specific CD8^+^ T-cell responses in every participant. The average magnitude of CD8^+^ T-cell responses per patient showed no statistically significant differences between the three groups of acute and resolved COVID-19 patients and seronegative controls. There were fewer NSP12-specific CD8^+^ T-cell responses with a lower magnitude compared to the CD4^+^ T-cell responses. The overall response pattern closely resembled that of the NSP12 CD4^+^ T-cell responses (**Supplementary Figure S2**). The location and patient-specific distribution of all individual NSP12-specific CD8^+^ T-cell responses are shown in **Supplementary Table S5**.

### 3.5 Longitudinal assessment of the NSP12-specific T-cell response before and after SARS-CoV-2 infection

The NSP12-specific peptide set elicited a similar breadth of responses in SARS-CoV-2-naive and SARS-CoV-2-exposed individuals—most likely because of the high preservation of this protein among Coronaviridae and therefore due to pre-primed CCC-specific T-cell responses. Therefore, in the next step, we also assessed the NSP12 peptide-specific response repertoire longitudinally, before and after a SARS-CoV-2 infection. For five patients, longitudinal samples (before and after contracting COVID-19) were available for further analysis.

The mean number of individual peptide-specific CD4^+^ T-cell responses detected before infection was 12.2 (range: 4–20) in these patients. This number increased only marginally and not significantly to 16.4 (range: 12–26) after SARS-CoV-2 infection (**Figure 2**). Patient HH-N12-37 even showed a decrease in responses after the COVID-19 infection (before: 20; afterward: 14).

Of note, each of the five individuals showed novel NSP12 peptide-specific CD4^+^ T-cell responses after COVID-19 infection (**Figure 2**). In **Supplementary Figure S3** and **Supplementary Table S4**, the detailed response repertoire and the distribution of NSP12-specific CD4^+^ T-cell responses are listed for each patient.

Patient HH-N12-34 recognized the highest number of peptide specificities (*n* = 7), which primed an NSP12 peptide-specific CD4^+^ T-cell response before and after SARS-CoV-2 infection. Overall, the NSP12 peptide-specific response repertoire only partially overlapped for the five individuals analyzed longitudinally at two time points, with the detection of new responses and loss of others—most likely CCC-specific T-cell responses—at the two different time points (31).

### 3.6 *In silico* analysis of the sequence similarity of SARS-CoV-2 NSP12 with CCC-specific peptides and serological evidence of previous exposure to CCCs

**Figure 3:**
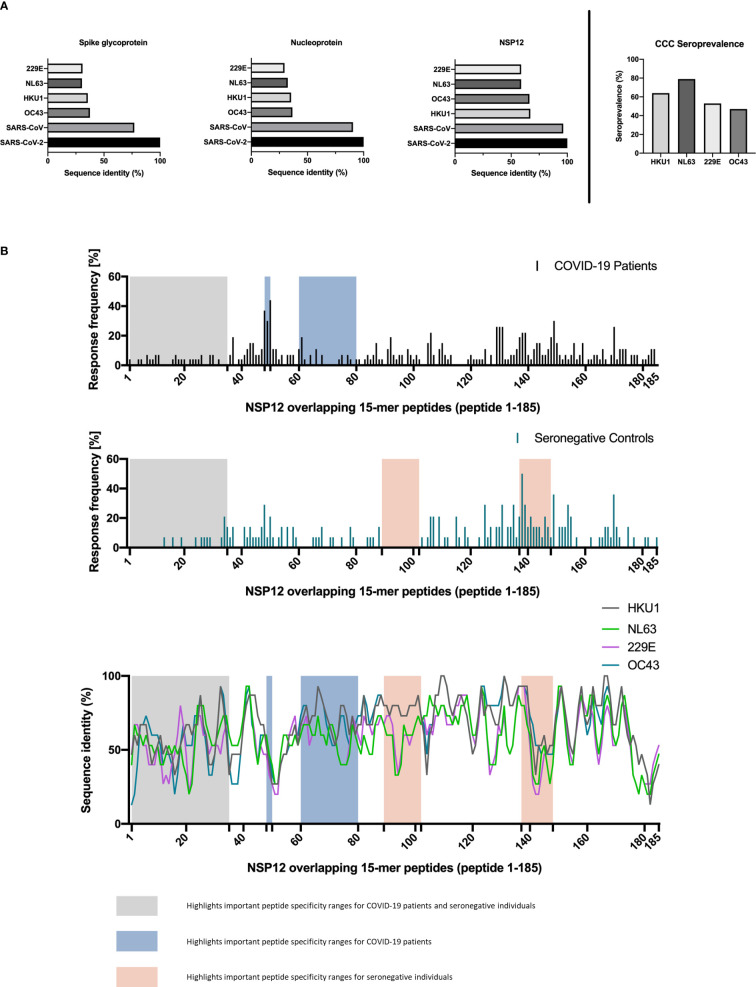
**(A, B)** Regions of SARS-CoV-2 NSP12 frequently targeted by seronegative individuals show different degrees of sequence identity with common cold coronaviruses. Canonical protein amino acid sequences of SARS-CoV-2 and common cold coronaviruses were extracted from the UniProtKB database and aligned using the UniProt blast tool. **(A)** The SARS-CoV-2 NSP12 protein has a higher sequence identity with the corresponding proteins of the 229E, NL63, OC43, and HKU1 common cold coronaviruses than the structural spike glycoprotein and nucleocapsid protein. Serologic positivity for common cold coronaviruses was highly prevalent in the study cohort. **(B)** High response rates to SARS-CoV-2 NSP12 peptides in SARS-CoV-2-infected individuals and seronegative individuals are not restricted to regions with higher than overall sequence identity.

In a first step, to investigate the degree of T-cell response cross-reactivity with the four circulating CCCs, we started by analyzing the sequence identity of the most frequently detected SARS-CoV-2 epitopes in this study with the most widely circulating CCCs: 229E, HKU1, OC43, and NL63 (**Figure 3**). The amino acid sequences of these CCCs corresponding to the NSP12 of SARS-CoV-2 showed a sequence identity genome homology of up to 67.1% (range: 58.8%–67.1%) (**Figure 3**).

To put this in context with the degree of sequence identity of the structural proteins between different Coronaviridae, we also aligned the SARS-CoV-2 amino acid sequences of the spike glycoprotein and the nucleoprotein with the corresponding CCC sequences. The SARS-CoV-2 spike glycoprotein and nucleoprotein showed a sequence similarity of up to 40.8% (range: 29.5%–40.8%) with the corresponding CCC sequences (**Figure 3**).

Next, we also determined CCC IgG serologies for ten COVID-19 patients and four seronegative individuals by performing the recomLine SARS-CoV-2 IgG as described earlier. Positive results above the cutoff value indicated a prior CCC infection (32). Each of the four SARS-CoV-2 seronegative patients with available CCC serology showed IgG antibodies against NL63.

The absence of nucleocapsid and spike antibodies indicated the SARS-CoV-2 seronegative status of all four included seronegative individuals. One of the seronegative patients showed IgG antibodies against each of the four CCCs (median: 2; range: 1–4). The 10 COVID-19 patients with available CCC serology data had at least IgG antibodies against two CCCs (median: 3.5; range: 0–4). The most often positively tested CCC in our cohort was NL63 (11 out of 14). Overall, the CCC with the fewest positive IgG antibody responses was OC43 (seven out of 14 patients) (**Figure 3**; **Table 5**).

**Table 5:** Available CCC and SARS-CoV-2 IgG serologies (LineBlot Assay) for COVID-19 patients and seronegative controls.

Patient ID	Human coronavirus 229E	Human coronavirus NL63	Human coronavirus OC43	Human coronavirus HKU1	SARS-CoV-2
HH-N12-8	☒	✓	✓	✓	✓
HH-N12-10	✓	✓	✓	✓	✓
HH-N12-11	☒	☒	☒	☒	✓
HH-N12-23	☒	✓	☒	☒	☒
HH-N12-27	☒	✓	☒	☒	☒
HH-N12-25	✓	✓	☒	✓	☒
HH-N12-12	☒	☒	☒	☒	✓
HH-N12-6	☒	☒	☒	✓	✓
HH-N12-1	✓	✓	✓	✓	✓
HH-N12-15	✓	✓	☒	☒	✓
HH-N12-26	✓	✓	✓	✓	☒
HH-N12-3	✓	✓	✓	✓	✓
HH-N12-17	✓	✓	✓	✓	✓
HH-N12-24	✓	✓	✓	✓	✓

In addition, we identified the three SARS-CoV-2 NSP12 peptide specificities with the highest response frequency in our seronegative cohort and their amino acid sequence identity between different human coronaviruses. (**Table 3**) The peptide specificity NSP12_138 (aa686–700) with the highest response frequency of 50% differed from corresponding sequences in the four CCCs by a mean of 1.5 amino acids (range: 1–3). The peptide specificities NSP12_149 (aa741–755) and NSP12_170 (aa846–860), both showing a response frequency of 36% in the seronegative cluster, differed by a mean of 5.13 amino acids (range: 4–7).

We compared the distribution response pattern of the peptide-specific CD4^+^ T-cell responses with respect to the location within the NSP12 in protein in COVID-19 patients and seronegative individuals. We also aligned the 15-mer amino acid sequences of the SARS-COV-2 peptides and corresponding CCC peptides to determine the sequence identity with NSP12 on a single-peptide level (**Figure 3**).

Two regions of the NSP12 with especially high response frequencies in COVID-19 patients were the peptide specificities NSP12_48–50 (aa236–260) and the peptide specificities NSP12_137–139 (aa681–705) in the seronegative controls. All study groups showed only low response rates for peptide specificities NSP12_1–35 (aa1–185).

Furthermore, the peptide specificities NSP12_60–80 (aa296–410) elicited only a low response rate in COVID-19 patients, and the peptide specificities NSP12_89–102 (aa441–520) elicited no response at all in individuals without exposure to SARS-CoV-2 (**Figure 3**). The single-peptide sequence identity for the four different CCCs is also shown in parallel to the depicted distribution of NSP12 CD4^+^ T-cell responses (**Figure 3**). The sequence analysis further revealed that the sequence identity did not exceed 60% for the peptide specificties NSP12 1-20 (aa1-110), which is a possible explanation for the low response frequency of seronegative controls in this area.

However, there were also areas such as the peptide specificities NSP12_140–148 (aa696–750) where, despite low sequence identity for all CCCs, a response rate of over 20% was observed for the seronegative controls. Peptide specificities NSP12_137–139 (aa681–705), which had the highest response rate for seronegative controls, showed a median amino acid sequence match of 93% (range: 67%–93%).

A detailed analysis of the distribution of NSP12-specific CD4^+^ T-cell responses in COVID-19 patients and seronegative individuals and the sequence similarity with CCCs over the different NSP12 regions revealed a broad overall distribution of the individual peptide-specific CD4^+^ T-cell responses across the entire protein. Peptides with high detection frequencies seemed to be located in areas with high genetic conservation of SARS-CoV-2 and high sequence identity with other CCCs.

### 3.7 Assessment of the *in vitro* cross-reactivity of CCC-specific peptide specificities in pre-pandemic samples with corresponding SARS-CoV-2 NSP12 peptide specificities

**Figure 4:**
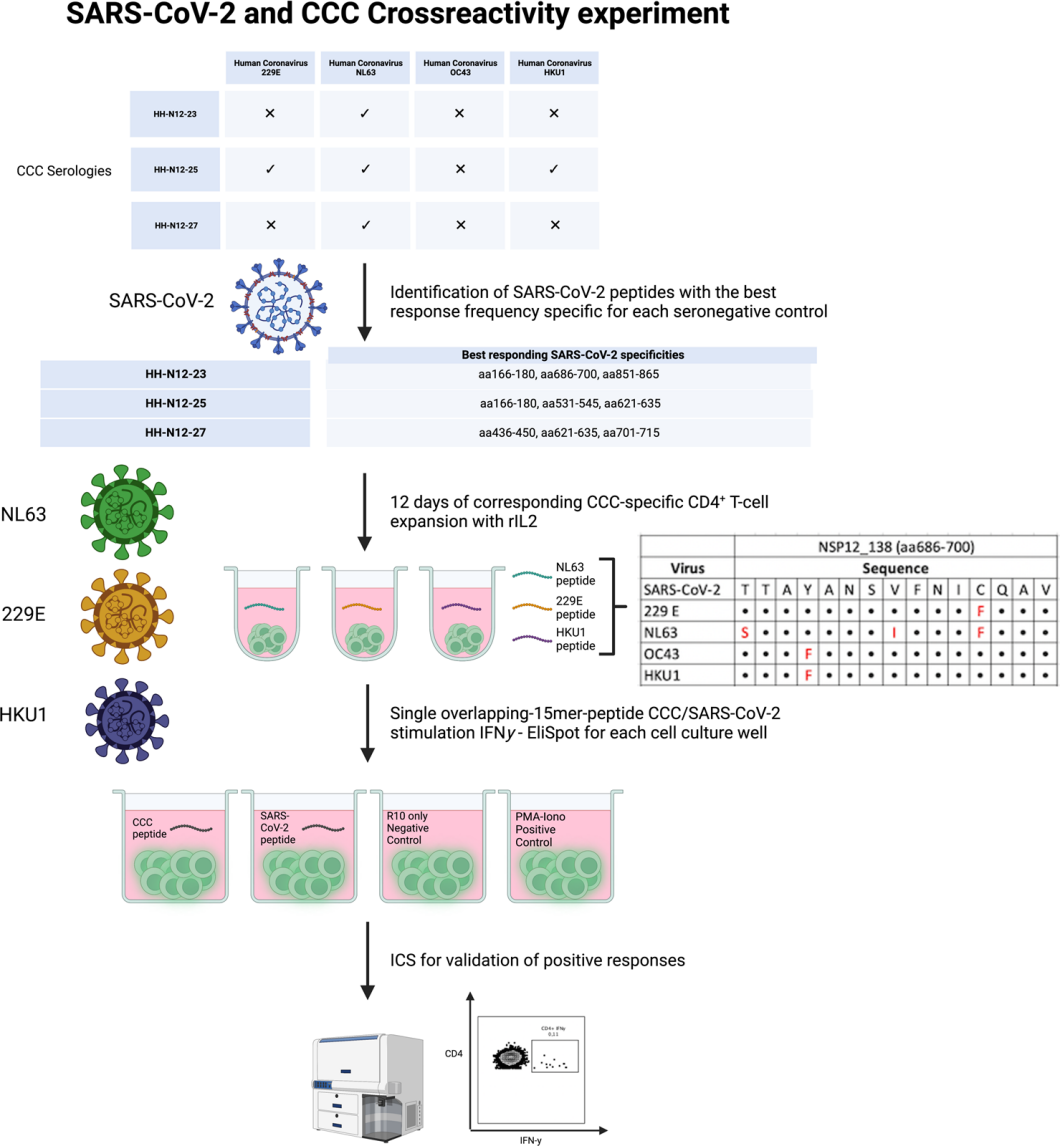
Experimental setup for CCC and SARS-CoV-2 T-cell cross-reactivity assessment.

PBMCs of three seronegative controls (HH-N12-23, HH-N12-25, and HH-N12-27) who had detectable NSP12-specific T-cell responses were subsequently stimulated *in vitro* with the different, individual corresponding CCC 15-mer peptide specificities. We then re-stimulated these antigen T-cell cultures after 12 days with either the CCC peptide or the respective SARS-CoV-2 peptide and performed IFN-γ-ELISpot and ICS for testing for the subsequent analysis of potential cross-reactivity (**Figure 4**).

**Figure 5:**
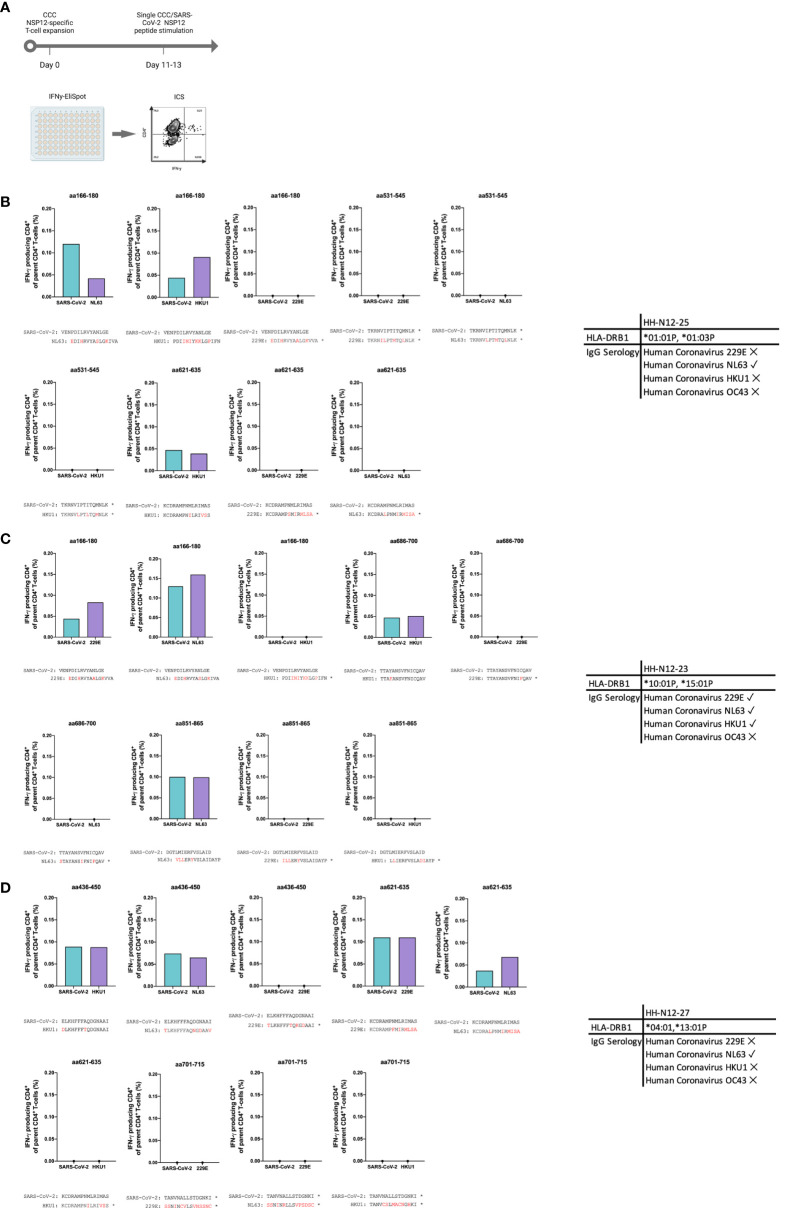
**(A–D)** *In vitro*-expanded common cold coronavirus NSP12-specific CD4^+^ T-cells of seronegative controls cross-recognize corresponding SARS-CoV-2 NSP12 peptides. **(A)** PBMC of *n* = 3 seronegative controls were expanded *in vitro* with common cold coronavirus NSP12 peptides (according to their serologic positivity) as described before. After 11-13 days, the cells were analyzed for IFN-γ after individual restimulation with single peptides with the sequence of common cold coronaviruses and the corresponding SARS-CoV-2 peptides. **(B–D)** In all three individuals, considerable cross-recognition to corresponding SARS-CoV-2 peptides could be detected despite amino acid differences at certain positions (the asterisk shows sequences from 15-mer peptides used for stimulation that did not elicit a CD4^+^ T-cell response in the IFN**-**γ-ELISpot).

In detail, the cross-reactivity was assessed for peptide-specific T cells of pre-pandemic samples directed against CCC-specific peptides compared to their SARS-CoV-2 peptide analogs. Cells were cultured with NL63, 229E, and HKU1 15-mer peptides in separate wells and restimulated after 11–13 days with the corresponding SARS-CoV-2 NSP12 15-mer peptides and the CCC 15-mer peptide in a different IFN-γ-ELISpot well. In the case of a positive IFN-γ-ELISpot response, an ICS was performed for the validation of the CD4^+^/CD8^+^ T-cell response (**Figure 5**).

Eight different NSP12-specific peptide specificities that had previously elicited CD4^+^ T-cell responses in at least two of the three seronegative samples were synthesized for the CCCs HKU1, NL63, and 229E. Each of the three individuals had serological evidence for exposure, defined as testing positive for IgG antibodies, against at least one of the three CCCs. Only the second individual (HH-N12-23) displayed antibodies against all three CCCs. In each of the three seronegative individuals, we detected specific CD4^+^ T-cell responses against the CCC peptide and the corresponding NSP12 SARS-CoV-2 peptide at a similar breadth (mean: 4; range, 3–5).

The amino acid sequences for both viruses are shown below each graph, but some peptide specificities did not show any response in the IFN-γ-ELISpot. They are labeled with an asterisk “*” (**Figure 5**). This experimental setup clearly demonstrates CD4^+^ T-cell cross-reactivity of several NSP12-specific 15-mer peptides with the corresponding CCC amino acid sequence-derived peptides and vice versa.

## 4 Discussion

Recently, we and others published in-depth characterizations of the breadth of the single-peptide-specific response directed against the spike glycoprotein and other structural SARS-CoV-2 proteins (N, M, E) using highly sensitive techniques (2, 3). In analogy to these studies, the current study provides a detailed dataset on the range and specificity of the T-cell response directed against the SARS-CoV-2 NSP12 (RdRp).

We detected a high response rate and a rather broad, low-level (33) NSP12-specific T-cell response. The majority of these NSP12 peptide-specific T-cell responses were CD4^+^ T-cell responses, as has been described for the T-cell response directed human COVID-19 T-cell response directed against other SARS-CoV-2 proteins (2, 3). We identified 160 out of 185 individual peptide specificities within this comprehensive overlapping peptide set that elicited an antigen-specific CD4^+^ T-cell response in at least one of the 41 patients. We also found low-magnitude CD8^+^ T-cell responses to a lesser extent in COVID-19 patients as well as in seronegative controls (34).

Previous investigations showed evidence for some degree of cross-reactivity between pre-primed CCC-specific T-cell responses and the SARS-CoV-2 response directed against the spike glycoprotein or the N, M, or E proteins (2, 3, 35–37). Furthermore, there are clinical observations that CCC seropositivity or the presence of cross-reactive T cells might affect the outcome of a subsequent SARS-CoV-2 infection (38). However, these results were not consistently found in all cohorts and are sometimes difficult to assess since the clinical outcome depends on a multitude of variables (39–42).

Importantly, the NSP12 is highly conserved between the different CCCs and SARS-CoV-2 (up to 67.1% sequence identity). SARS-CoV-1 even shows an NSP12 sequence identity of 96.4% with SARS-CoV-2 (43). Also, the NSP12 is highly conserved between the different variants of concern (VoC) and lineages under monitoring (LuM) of SARS-CoV-2 (44, 45). In our current study, 12 out of 14 patients had positive IgG antibody titers for at least one CCC.

Remarkably, the number of NSP12-specific CD4^+^ T-cell responses was only slightly lower in the samples of individuals without a history of SARS-CoV-2 or pre-pandemic samples compared to COVID-19 patients, indicating an extremely high degree of cross-reactivity of CCC- and SARS-CoV-2 NSP12-specific T-cell responses.

Detailed sequence analysis and further *in vitro* experiments confirmed that some of the CCC and SARS-CoV-2 NSP12 epitopes had identical sequences, and other peptide sequences only differed by one or two amino acids and showed a high cross-reactivity *in vitro*. These results are in agreement with the recent findings of other studies (46, 47) that identified specific and cross-reactive CD4^+^ T-cell epitopes in the CCC OC43 genome. The impact of evolving SARS-CoV-2 variants on the antigen-specific T-cell response is subject to extensive further research (48–50).

Previously, we and others showed that the magnitude but not the number of spike-specific peptide responses increased after repeated vaccination with mRNA spike glycoprotein vaccines (3, 51). This result is not unexpected, as a pre-primed spike- or CCC-specific T cells will expand upon re-encounter with the variant spike or NSP12 antigen according to the theory of antigenic imprinting (31, 52).

However, the interferences between SARS-CoV-2 and the other four CCCs, each having different epidemiology, tropism, and antigenicity, seem to be more complex (34, 53). Lately, it has also been reported that exposure to SARS-CoV-2 might interfere with CCC responses, either directly or indirectly. CD4^+^ T-cell responses against CCCs are both increased and decreased in COVID-19 patients.

Of note, a number of other viruses also seem to exhibit some structural similarities with SARS-CoV-2 (54–56). The relevance of these potential cross-reactivities for the clinical course of infection and immunological parameters is yet to be investigated in detail. Similarly, while we saw a high level of cross-virus reactivity between CCC and the corresponding SARS-CoV-2-NSP12 epitopes, the picture was much more heterogeneous in the longitudinal analysis of five CCC seropositive individuals.

While we detected slightly more NSP12-specific responses after SARS-CoV-2 infection, this difference was not statistically significant. Also, the response repertoire changed over time—some responses decreased in magnitude or could not be detected again. It has to be taken into account that, methodically, (A) we analyzed T cells, not *ex vivo* but after short-term expansion; (B) the patient’s histories differed in terms of past infections as well as the SARS-CoV-2 course of infection; and (C) the timing of sampling after infection differed.

The individual HLA haplotype and even the timing and kinetics of the NSP12 antigen processing should be considered being highly heterogeneous. No broader conclusions can be drawn from this small patient cohort about the clinical implications of the detected cross-reactivities. However, there is a clear interdependence that is highly complex, might differ from patient to patient depending on the exact previous exposure to different antigens, and is—judged on its own—of yet unclear clinical significance (40).

The current study has several limitations: firstly, our seronegative controls could have theoretically been exposed to SARS-CoV-2 despite a negative history of acute viral illness and negative serology for nucleocapsid antibodies (NC-Abs) (57, 58), since it has been described that NC-Abs are sometimes not primed after infection or decline below the limit of detection in some cases (59, 60). However, the seronegative controls in the current study were recruited early during the pandemic, and nine out of nine seronegative controls later exhibited a COVID-19 infection with a positive SARS-CoV-2 PCR result and NC seroconversion in the highly sensitive and specific routine immunoassay [see the **Materials and methods** section (61)]. Of note, the profile of the NSP12-specific T-cell responses did not differ between the pre-pandemic and seronegative individuals. Secondly, the NSP12-specific responses were assessed only after NSP12-specific *in vitro* expansion in polyclonal cell cultures, which to some extent limits the comparability with *ex vivo* or *in vivo* settings. It might be influenced by stochastic effects due to the preferential expansion of certain peptide-specific clones when expanding with peptide mixes. Potentially, our results are biased by the study design, in which we used overlapping 15-mer peptides and one epitope might be located in between two adjacent peptides. Also, our methodology might bias toward the detection of NSP12 CD4^+^ T-cell responses.

Also, the exact CCC infection history of the study participants (some of whom were also immunosuppressed) is not known, and we were also only able to obtain the CCC serologies of a subset of individuals. Additionally, the CCC-serological response can wane over time (62, 63), and a previous exposure might have been missed by just relying on the current serological test applied (64, 65). Furthermore, the autologous sequences of any of the respective infecting viruses are not known. Lastly, the assessment of the samples was not standardized in terms of sample acquisition and time after the SARS-CoV-2 infection.

Larger prospective studies need to assess (A) the impact of the T-cell response directed against structural proteins versus nonstructural proteins on the clinical course of a SARS-CoV-2 infection and as potential vaccination antigens; (B) the overall influence of positive CCC serology (and previous CCC infection) on disease outcome and certain T-cell responses; as well as (C) the influence of pre-pandemic imprinting on T-cell responses after a COVID-19 infection on the clinical course of other subsequent CCC infections and vice versa (66, 67).

Also, the *ex vivo* phenotype and *ex vivo* functionality of the NSP12-specific T-cell response in different tissues and after vaccination with inactivated whole virus vaccines (that include NSP12 as antigen) will be of interest.

Further research with newly developed methods (68, 69) could help to extend the results of this current study. The validation of our results in the context of complex antigens is needed (70). Also, the analysis of other NSPs of SARS-CoV-2 could prove useful for assessing the validity and relevance of these findings (71). The current study will be a useful resource for the development of novel NSP12 MHC class II tetramers (72) and provide further insight into the possibility of NSP12-based vaccines (73, 74).

This comparative high-resolution analysis of immunodominant NSP12 single-peptide T-cell specificities in COVID-19 patients with different infections and HLA backgrounds is evidence of the complexity and interdependence between pre-primed CCC T-cell responses and those directed against SARS-CoV-2 (7, 10).

In summary, we present a detailed investigation into the breadth of the single-peptide NSP12 CD4^+^ T-cell response in a cohort of COVID-19 patients with known HLA backgrounds. We find a uniformly low-level, broadly directed T-cell response with several frequently detected NSP12 peptide specificities both in COVID-19 patients and SARS-CoV-2-naive individuals.

Simultaneously, we find evidence using sequence comparison, pre-pandemic samples, and *in vitro* experiments for a high degree of cross-reactivity of these responses with pre-primed CCC-specific responses. Only acutely infected SARS-CoV-2 patients show a significantly higher magnitude of the NSP12-specific T-cell response compared to SARS-CoV-2 seronegative individuals.

## Data availability statement

The raw data supporting the conclusions of this article will be made available by the authors, without undue reservation.

## Ethics statement

The studies involving human participants were reviewed and approved by Ärztekammer Hamburg (PV4780, PV7298). The patients/participants provided their written informed consent to participate in this study.

## Author contributions

TW: Conceptualization; data curation; formal analysis; investigation; methodology; visualization; writing – original draft; writing – review and editing. MM: Conceptualization, investigation; methodology, project administration, supervision. HK: Conceptualization; data curation; investigation; methodology; writing – review and editing. LC: Data curation; investigation; methodology; visualization; writing – original draft. MK: Data curation; investigation; visualization. SS: formal analysis; investigation; methodology. LH: Data curation; resources. SPe: Resources. VD: Resources. AG: Resources, Validation. MA: Resources. SH: Resources, Validation. AS: Resources, Validation. ML: Resources. SPi: Resources. WK: Resources; validation. JS: Resources; validation. JSzW: Conceptualization; funding acquisition; methodology; project administration; resources; supervision; writing – original draft; writing – review and editing. All authors contributed to the article and approved the submitted version.

## Supplementary material

Supplementary Figure 1 Representative gating strategy.

Supplementary Figure 2
**(A–C)** NSP12-specific CD8^+^ T-cells responses. **(A)** Number of individual NSP12-peptide-specific CD8^+^ T-cell responses and average magnitude per individual of IFN-y producing CD8^+^ T-cells. **(B)** Representative flow cytometry plots of HH-N12-38. **(C)** Distribution of SARS-CoV-2 NSP12 CD8^+^ T-cell responses in COVID-19 patients and seronegative individuals on a single 15-mer peptide level.

Supplementary Figure 3 Longitudinal characterization of the distribution and magnitude of NSP12-specific CD4^+^ T-cell responses of each individual (n=5) before and after COVID-19 infection.
