# Supplementary figures and images for: Evidence for broad cross-reactivity of the SARS-CoV-2 NSP12-directed CD4+ T-cell response with pre-primed responses directed against common cold coronaviruses
Source: Front Immunol. 2023 May 5;14:1182504. doi: 10.3389/fimmu.2023.1182504 (PMC10196118; doi:10.3389/fimmu.2023.1182504)

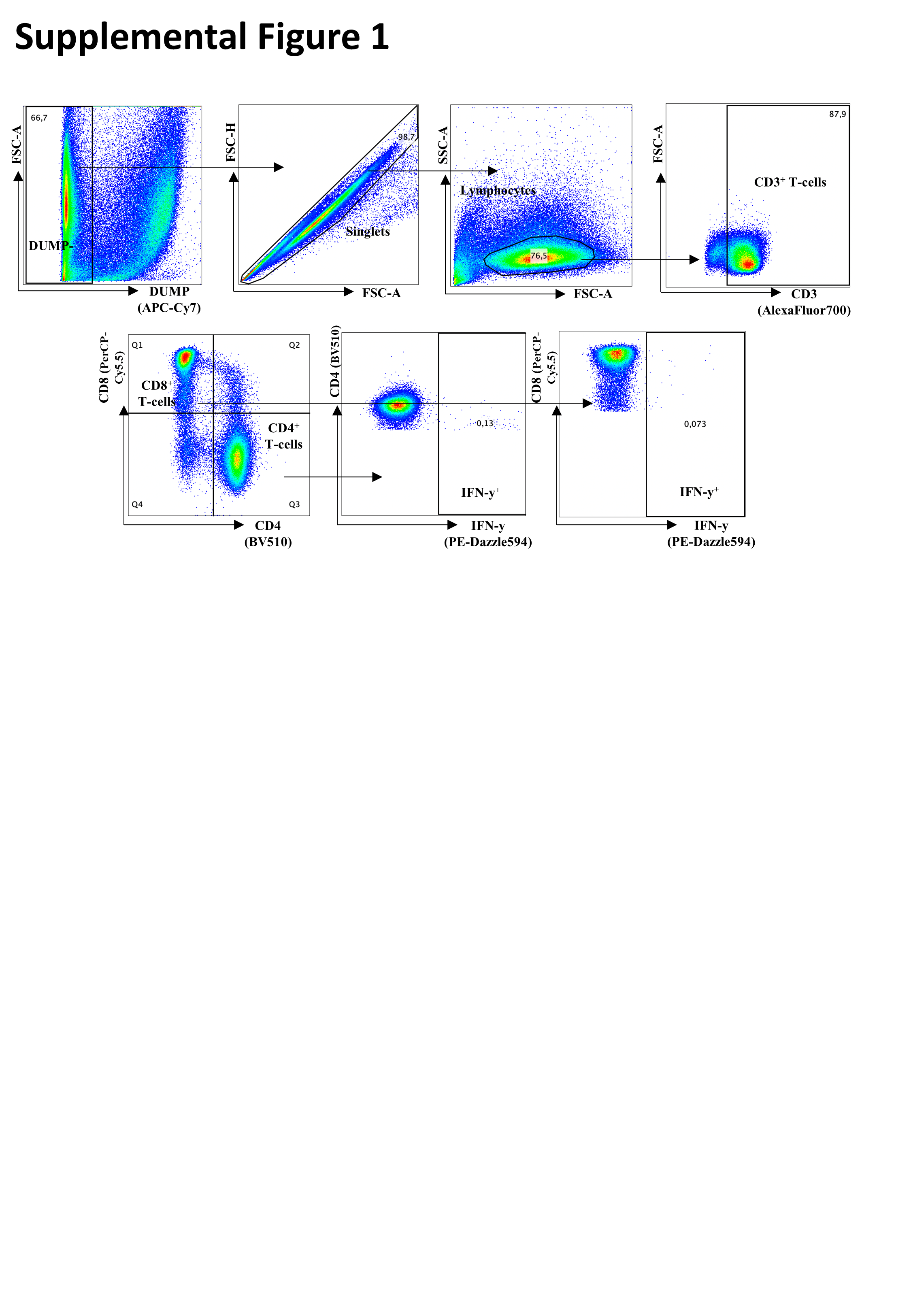

Supplement: Supplementary Figure 1 — Representative gating strategy. [file Image_1.tif]

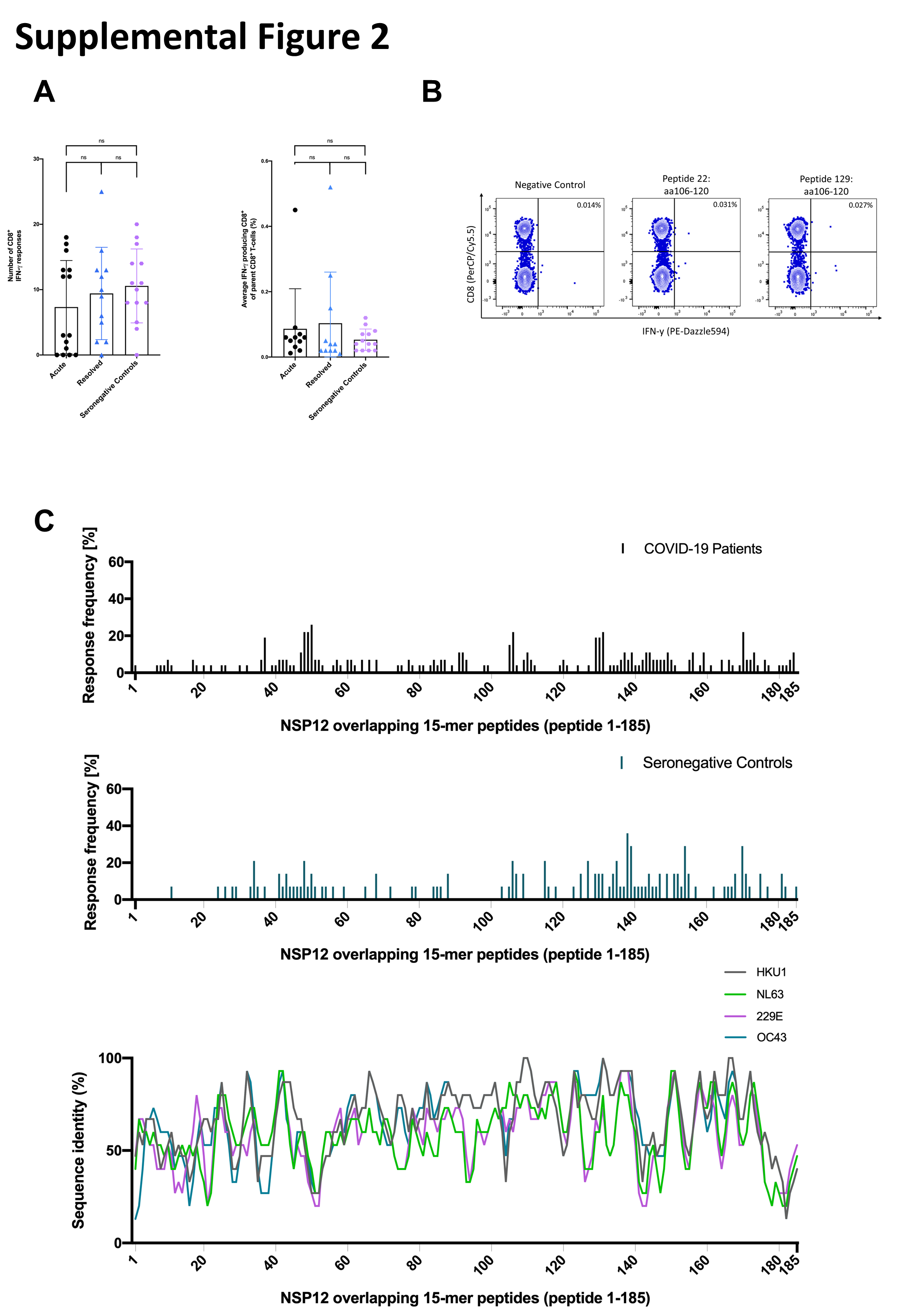

Supplement: Supplementary Figure 2 — (A–C) NSP12-specific CD8+ T-cells responses. (A) Number of individual NSP12-peptide-specific CD8+ T-cell responses and average magnitude per individual of IFN-y producing CD8+ T-cells. (B) Representative flow cytometry plots of HH-N12-38. (C) Distribution of SARS-CoV-2 NSP12 CD8+ T-cell responses in COVID-19 patients and seronegative individuals on a single 15-mer peptide level. [file Image_2.tif]

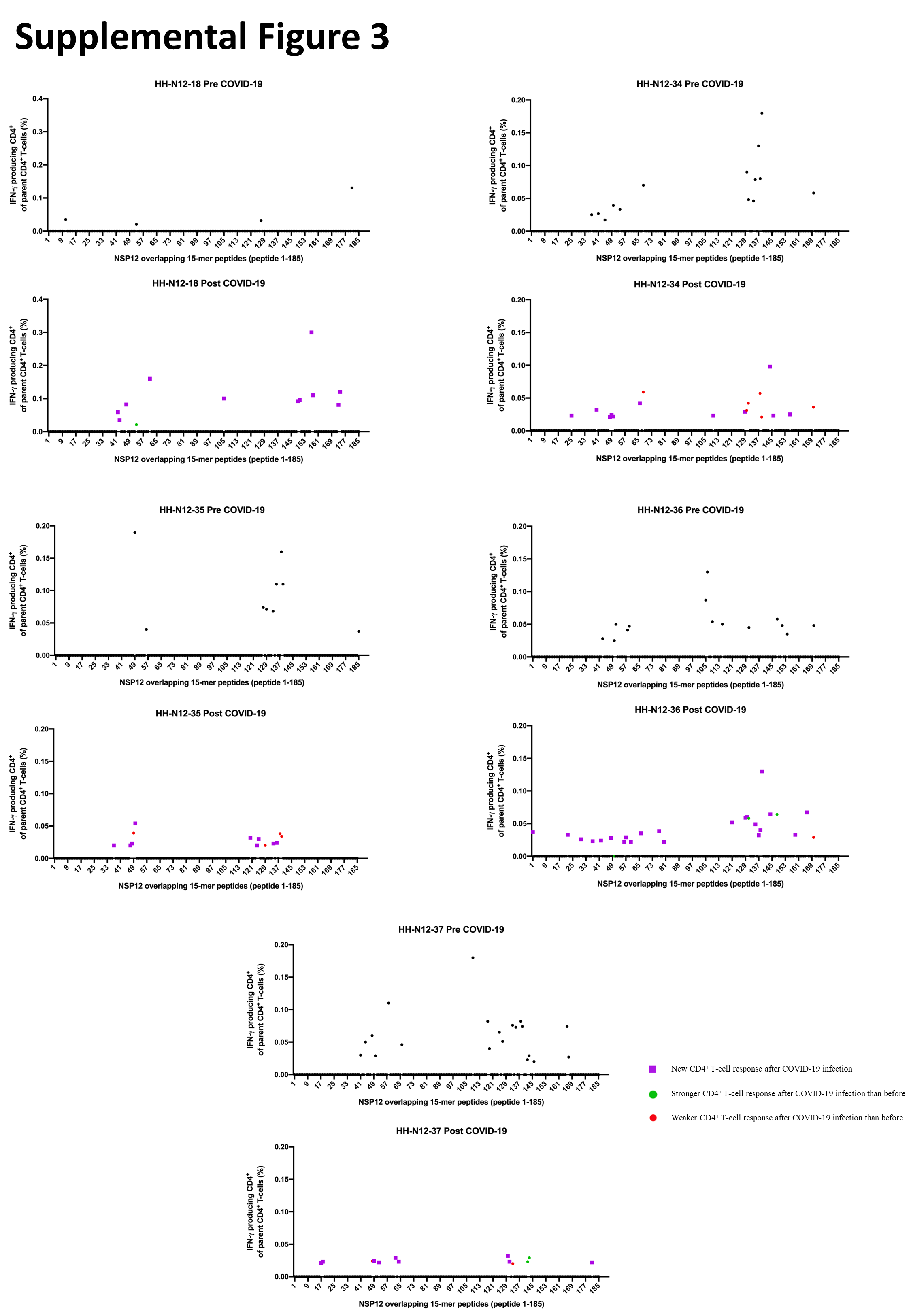

Supplement: Supplementary Figure 3 — Longitudinal characterization of the distribution and magnitude of NSP12-specific CD4+ T-cell responses of each individual (n=5) before and after COVID-19 infection. [file Image_3.tif]
